# Plasmid interference for curing antibiotic resistance plasmids *in vivo*

**DOI:** 10.1371/journal.pone.0172913

**Published:** 2017-02-28

**Authors:** Muhammad Kamruzzaman, Shereen Shoma, Christopher M. Thomas, Sally R. Partridge, Jonathan R. Iredell

**Affiliations:** 1 Centre for Infectious Diseases and Microbiology, The Westmead Institute for Medical Research, The University of Sydney, Westmead, New South Wales, Australia; 2 Institute of Microbiology and Infection, University of Birmingham, Birmingham, United Kingdom; 3 Westmead Hospital, Westmead, New South Wales, Australia; University of Manchester, UNITED KINGDOM

## Abstract

Antibiotic resistance increases the likelihood of death from infection by common pathogens such as *Escherichia coli* and *Klebsiella pneumoniae* in developed and developing countries alike. Most important modern antibiotic resistance genes spread between such species on self-transmissible (conjugative) plasmids. These plasmids are traditionally grouped on the basis of replicon incompatibility (Inc), which prevents coexistence of related plasmids in the same cell. These plasmids also use post-segregational killing (‘addiction’) systems, which poison any bacterial cells that lose the addictive plasmid, to guarantee their own survival. This study demonstrates that plasmid incompatibilities and addiction systems can be exploited to achieve the safe and complete eradication of antibiotic resistance from bacteria *in vitro* and in the mouse gut. Conjugative ‘interference plasmids’ were constructed by specifically deleting toxin and antibiotic resistance genes from target plasmids. These interference plasmids efficiently cured the corresponding antibiotic resistant target plasmid from different *Enterobacteriaceae in vitro* and restored antibiotic susceptibility *in vivo* to all bacterial populations into which plasmid-mediated resistance had spread. This approach might allow eradication of emergent or established populations of resistance plasmids in individuals at risk of severe sepsis, enabling subsequent use of less toxic and/or more effective antibiotics than would otherwise be possible, if sepsis develops. The generalisability of this approach and its potential applications in bioremediation of animal and environmental microbiomes should now be systematically explored.

## Introduction

The efficacy of antibiotics used for decades to treat serious infections is increasingly threatened by large, low-copy, self-transmitting resistance plasmids in the *Enterobacteriaceae*. These conjugative plasmids are arguably the most important vectors of modern antibiotic resistance, and are directly linked to major outbreaks of antibiotic resistant infection [1–4]. Modern resistance plasmids may spread through a range of different member species of the *Enterobacteriaceae* as a plasmid epidemic, but host strain contributions to plasmid-encoded antibiotic resistance phenotypes [4] further complicate surveillance and control [1, 4]. A conjugative resistance plasmid in the microflora directly increases risk of therapeutic failure [5], and may spread resistance to others. Antibiotic resistance carried on large conjugative plasmids may also persist for months even in the absence of ongoing specific selection [6]. Even when spread of a particular resistance plasmid is defined early enough for implementation of containment strategies, the only available option is to use an antibiotic to which the plasmid does not confer resistance, in an attempt to entirely eliminate all bacterial populations that carry it.

Older antibiotics and those not used as primary therapy in severe sepsis may provide options for killing bacterial populations harbouring dangerous plasmids. Indeed, non-absorbable antibiotics such as colistin and neomycin have long been used to ‘selectively decontaminate’ the gut. The consequences of this ablative approach are not fully defined and is not widely adopted despite promising results in clinical trials [7, 8], due to clinician concerns about development of antibiotic resistance [9, 10]. Approaches that specifically eradicate problem plasmids and the phenotypes they encode without destroying host bacterial populations or other resident plasmids is the ideal next step toward microbial husbandry.

Multiple plasmids commonly coexist in the same bacterial cell but cross-interference between plasmid replication systems ensures that the most closely related plasmids are incompatible and cannot stably persist together [11, 12]. Entry exclusion systems (EES) also inhibit conjugation of a plasmid into a cell that already has a resident plasmid of the same ‘exclusion group’ by ten- [13] to more than a thousand-fold under certain conditions [14]. Strong selection for a plasmid entering a bacterial population therefore normally results in displacement of any resident incompatible plasmids that are not selected, allowing the incoming plasmid to take over the ecological niche.

**Fig 1:**
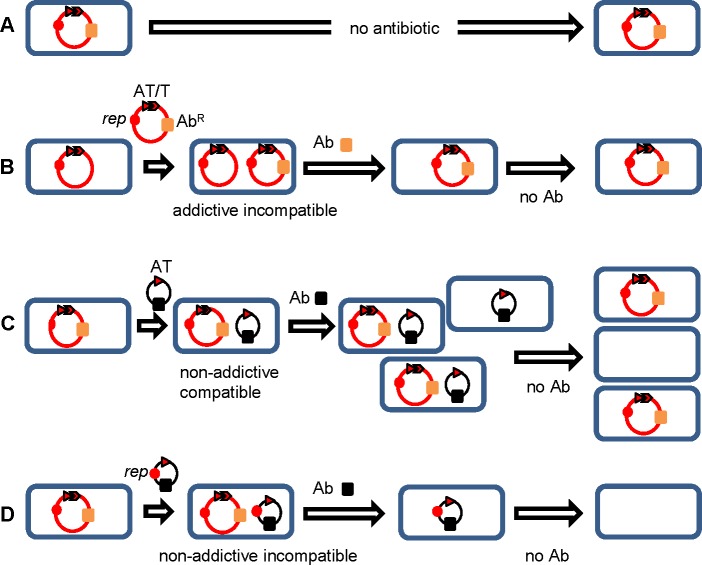
Addictive antibiotic resistance plasmids. The replicon (*rep*, solid circle), antitoxin (AT, arrowhead) and toxin (T, arrow) genes of a PSK/addiction system, an antibiotic resistance gene (Ab^R^) and corresponding antibiotic (Ab, solid blocks) are shown. (**A**) An addictive plasmid is stable in the absence of antibiotic selection. (**B**) An addictive plasmid can be displaced by an incompatible plasmid. (**C**) A compatible plasmid providing specific antitoxin (non-addictive compatible) leads to loss of addictive resistance plasmids from some cells. (**D**) An incompatible non-addictive interference plasmid providing specific antitoxin (non-addictive incompatible) ensures that all bacterial cells are ultimately free of both plasmid types.

Large conjugative low-copy number plasmids have also acquired specific ‘addiction’ mechanisms that are important for their long term persistence (Fig 1). Small protein toxins that help regulate bacterial death under stress conditions were first identified as part of post-segregational killing (PSK) / ‘addiction’ systems [15] in plasmids. A typical addiction system includes a long-lived toxin and cognate short-lived antitoxin, with the unopposed toxin killing any bacteria that have lost the plasmid, and thus the encoded antitoxin, during cell division [16–19] (Fig 1).

T/A systems are generally classified by mode of action with the largest numbers and diversity in types I and II. In type I systems, e.g. *pndBCA* found in IncI1 plasmids, toxin (PndA) translation is inhibited by an unstable antisense RNA (*pndB*) transcribed in reverse orientation at the toxin gene locus [20–22]. In type II systems e.g. *pemIK*, first described in the IncFII plasmid R1 as Kis-Kid [23] and common in IncL/M and IncF plasmids, the protein antitoxin (PemI) is stable only when complexed with the cognate toxin PemK [24, 25].

The combined effects of addiction, incompatibility and repeated antibiotic exposure favour the emergence of a few lineages of resistance plasmids in populations of antibiotic resistant *Enterobacteriaceae* [26], possibly at the expense of diverse antibiotic-susceptible indigenous plasmids that have evolved over thousands of years. Targeting of incompatibility and addiction systems to selectively remove plasmids from bacterial populations has been proposed as part of an optimal ecological/evolutionary approach [27] that had been previously demonstrated *in vitro* with IncP and IncF plasmids [28]. Conjugative addictive plasmids (including plasmids with multiple addiction systems) can also be eliminated *in vitro* by blocking replication, with [28] or without [29, 30] blocking addiction, by using intercalating dyes and drugs such as quinolones [31], small molecule replication inhibitors [32, 33] and even by applying heat stress [34], while fatty acids or tanzawaic acids can be used to inhibit spread by conjugation [35, 36] but no such approach has ever been demonstrated *in vivo*.

We postulated that antibiotic susceptibility could be restored to an entire gut microbiome by the introduction of an appropriately-designed, orally administered conjugative ‘interference’ plasmid without the gain of any new antibiotic resistance and without the loss of the original bacterial populations or of any bystander plasmid populations. Plasmids encoding resistance to carbapenems and/or third-generation cephalosporins, which often also carry aminoglycoside resistance determinants, are the highest priority for elimination. Two locally-endemic plasmids carrying different β-lactamase genes and with different replicons and different addiction systems were therefore chosen for an experimental proof of principle.

## Materials and methods

### Bacteria, culture conditions, primers and plasmids

Tables 1 and 2 and S1 Table list plasmids, bacterial strains and primers, respectively. Bacteria were grown in Luria-Bertani (LB) broth (Invitrogen, CA, USA) and plated on CHROMagar Orientation (CHROMagar, Paris, France). Ampicillin (100 μg/mL), tetracycline (10 μg/mL), gentamicin (8 μg/mL), chloramphenicol (20 μg/mL), cefotaxime (8 μg/mL), fosfomycin (200 μg/mL), rifampicin (100 μg/mL), and/or sodium azide (100 μg/mL) were added as indicated. Chemical transformation and electroporation were carried out using standard protocols. Conjugations were performed by filter mating [37], with overnight or 2 h incubation on filters before antibiotic selection of specific transconjugants. Conjugation efficiency was calculated as the number of transconjugants per donor cell.

**Table 1:** Plasmids used in this study.

Plasmid	Characteristics	Source/Reference
pBCSK+	High copy phagemid cloning vector (CHL^R^)	Stratagene, USA;
pGEM-T Easy	Cloning vector for direct cloning of PCR products (AMP^R^)	Promega, USA
pBAD18	Arabinose inducible expression vector (AMP^R^)	[38]
pKM200	Plasmid carrying the lambda-Red recombinase system (CHL^R^)	Addgene, USA
pEl1573	Naturally occurring conjugative IncL/M plasmid carrying *bla*_IMP-4_ from clinical isolate *E*. *cloacae* El1573 (JX101693)	[39, 40]
pJIBE401	Naturally occurring conjugative IncL/M plasmid from clinical isolate *K*. *pneumoniae* Kp1239; identical to pEl1573	[39]
pJIE512b	Naturally occurring conjugative IncI1 plasmid carrying *bla*_CMY-2_ from clinical isolate *E*. *coli* JIE512b (HG970648)	[22]
pJIMK3	*pemI* gene of pEl1573 in *Sma*I site of pBCSK+	This study
pJIMK21	IncL/M *rep* genes of pEl1573 in pGEM-T Easy	This study
pJIMK25	*Not*I L/M *rep* fragment from pJIMK21 in *Not*I site of pJIMK3	This study
pJIMK39	*pemI* and IncL/M *rep* from pJIMK25 in *Sma*I site of pBAD18	This study
pJIMK41	Construct to replace pEl1573 *pemK* with *fosA3*	This study
pJIMK43	Construct to replace pEl1573 MRR with *tetA*	This study
pJIMK45	pEl1573 with ~28.5 kb including entire MRR replaced by *tetA*	This study
pJIMK46	pJIMK45 with part of *pemK* replaced by *fosA3*	This study
pJIMK50	Construct to replace pJIE512b *bla*_CMY-2_ with *fosA3*	This study
pJIMK54	pJIE512b with *bla*_CMY-2_ replaced by *fosA3*	This study
pJIMK55	Construct to replace pJIE512b *pndA* with *tetA*	This study
pJIMK56	pJIMK54 with part of *pndA* replaced by *tetA*	This study

**Table 2:** Bacterial strains used in this study.

Bacteria	Characteristics	Source/Reference
DH5α	*E*. *coli* K-12; F‐, 80*lac*ZΔM15 Δ(*lac*ZYA‐*rg*F)U169 *deo*R, *rec*A1, *end*A1, *hsd*R17(rk‐mK+), *pho*A, *sup*E44, λ‐*thi*‐1, *gyr*A96, *rel*A1	Invitrogen (USA)
UB5201Rf	Rifampicin resistant *E*. *coli* K-12, F ‐, *pro*, *met*, *rec*A56, *gyr*A	[4]
J53Azi^r^	Azide resistant *E*. *coli* K-12, F-, *lac*+, *pro*, *met*	[41]
El1573	Multi‐drug resistant *E*. *cloacae* carrying pEl1573	[39]
Kp1239	Multi‐drug resistant *K*. *pneumoniae* carrying pJIBE401	[4]
JIE512b	Multi-drug resistant *E*. *coli* carrying pJIE512b	[22]
Kp13883Rf	Rifampicin resistant derivative of *K*. *pneumoniae* ATCC13883	[4]
Mm1585Rf	Rifampicin resistant derivative of *Morganella morganii* Mm1585	[4]
Cf4000Rf	Rifampicin resistant derivative of *Citrobacter freundii* Cf4000	[4]

### PCR amplification and cloning

Platinum *pfx* DNA polymerase (Invitrogen, USA) was used to amplify blunt-ended PCR products. All PCR products were purified (PureLink Quick PCR Purification Kit; Invitrogen, USA). PCR and sequencing was used to confirm all constructs.

### Construction of specific plasmids

The antitoxin gene *pemI* with its own promoter and ribosome binding site (RBS) was amplified from pEl1573 as a blunt-ended PCR product and cloned into the unique *Sma*I site of pBCSK+ (CHL^R^; Stratagene, USA) to construct pJIMK3. The IncL/M replication genes (*repCBA*) were amplified from pEl1573 and cloned into pGEM-T Easy (Promega, USA) to construct pJIMK21. The *Not*I fragment from pJIMK21 containing *repCBA* was cloned into the unique *Not*I site of pJIMK3 to construct pJIMK25. *pemI*-*repCBA* was amplified from pJIMK25 and the blunt-ended product cloned into the unique *Sma*I site of pBAD18 (AMP^R^) to construct pJIMK39.

*E*. *coli* DH5α (β-galactosidase-negative, white on CHROMagar) carrying pJIMK39 (AMP^R^ CTX^S^) was mated with *E*. *coli* UB5201Rf (β-galactosidase-positive, pink on CHROMagar) carrying pEl1573 (AMP^R^ CTX^R^). Six white *E*. *coli* DH5α transconjugants picked from CHROMagar containing CTX were all confirmed to contain both pJIMK39 and pEl1573 (AMP^R^ CTX^R^) by PCR. After incubation (4 h, 37°C, 220 rpm) in LB broth containing AMP plus either glucose or arabinose (0.2% w/v) and subculture on antibiotic-free CHROMagar, 12 colonies from each of the 12 subcultures were screened for *bla*_IMP-4_ by PCR.

### Construction of conjugative interference plasmids

The *fosA3* fosfomycin resistance gene (e.g. GenBank accession no. JF411006) from *E*. *coli* 78AJTi [42] and the *tetA* tetracycline resistance gene (94% identical to *tetA*(A)) from plasmid N3 (FR850039) were each amplified with their native promoter and RBS. Short regions upstream and downstream of the regions to be replaced were amplified using primers overlapping with *tetA* or *fosA3* specific primers. Fusion products from Gibson assembly PCR [43] of the three amplicons (1:1:1 molar ratio) were cloned into pGEM-T Easy for use as templates to amplify larger amounts. Amplicons (~1.0 μg) were electroporated into UB5201Rf carrying pJIBE401 or J53Azi^r^ carrying pJIE512b, both also containing pKM200 encoding lambda Red recombinase [44]. Homologous recombination (at 30°C) was used to replace the target region with the antibiotic resistance marker, with subsequent growth at 37°C to remove pKM200. Colonies were selected on CHROMagar containing appropriate antibiotics and the replacements confirmed by PCR and sequencing.

**Fig 2:**
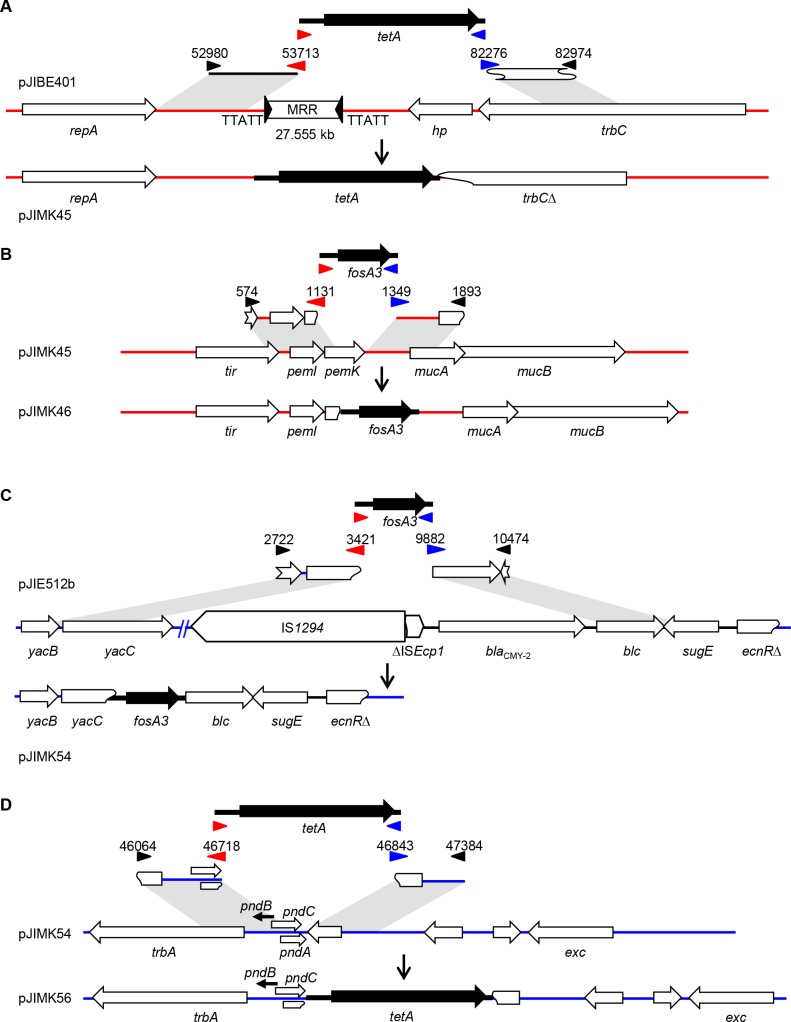
Construction of conjugative interference plasmids. pJIMK46 (**A**, **B**) was constructed from pJIBE401 by replacing 28.5 kb including the MRR with *tetA* and then part of the *pemK* toxin gene with *fosA3*. pJIMK56 (**C**, **D**) was constructed from pJIE512b by replacing the *bla*_CMY-2_ gene and flanking IS with *fosA3* and then part of the *pndA* toxin gene with *tetA*. Numbers indicate the positions of the amplified regions in GenBank accession nos. JX101693.1 (pEl1573) or HG970648.1 (pJIE512b). Blue and red arrows indicate overlapping primers, black arrows indicate other primers.

For pJIBE401, a 734 bp region upstream and a 699 bp region (including part of the *trbC* gene) downstream of the antibiotic multi-resistance region (MRR) were amplified for Gibson PCR with *tetA* to give pJIMK43. This was used to replace the entire 27.555 kb MRR of pJIBE401 (containing *bla*_IMP-4_) plus ~1.0 kb of flanking sequence to create pJIMK45 (Fig 2A). A 558-bp region including 116 bp of *pemK* and a 545-bp region immediately downstream of *pemK* were amplified from pJIBE401 for Gibson PCR with *fosA3* to give pJIMK41. This was used to replace 217 bp of the 333 bp *pemK* toxin gene of pJIMK45 to create pJIMK46 (Fig 2B). pJIBE401 (sequenced here) is identical to pEl1573 (JX101693) except for a single C→T change at position 60364 in IS*CR1*, which is in the antibiotic resistance region that was deleted to construct pJIMK46.

For pJIE512b a 700 bp region (part of the *yacBC* hypothetical genes) upstream and a 593 bp region (*blc* and part of *sugE*) downstream of *bla*_CMY-2_ were amplified for Gibson PCR with *fosA3* to give pJIMK50. This was used to replace a 6.460 kb region containing hypothetical proteins, IS*1294*, the truncated IS*Ecp1* and *bla*_CMY-2_ (pJIMK54; Fig 2C). A 655 bp region (218 bp of *trbA*, all of *pndC*) upstream and a 542 bp region downstream of *pndBCA* were amplified from pJIE512b for Gibson PCR with the *tetA* amplicon to give pJIMK55. This was used to replace the last 7 bp of the *pndA* toxin gene of pJIMK54 to give pJIMK56 (Fig 2D).

pJIBE401, pJIMK46 and pJIMK56 DNA was purified (HiSpeed plasmid midikit; Qiagen, Germany) and 1 ng of each used for library preparation (Nextera XT DNA sample preparation kit; Illumina, Inc., USA), with each of the three libraries indexed for sequencing (Illumina MiSeq; Australian Genome Research Facility, Melbourne, Australia). Geneious V7 (Biomatters, New Zealand) was used to map raw reads from pJIBE401 and pJIMK46 against pEl1573 (JX101693) and those from pJIMK56 against pJIE512b (HG970648).

### Mouse experiments

All research and animal care procedures were approved by the Animal Ethics Committee of the Western Sydney Local Health District (protocol 4205.06.13) in accordance with the ‘Australian Code of Practice for the Care and Use of Animals for Scientific Purposes’. Five week old female BALB/c mice (Animal Resource Centre; Perth, WA, Australia) were housed in groups of three in open-lid M1 polypropylene cages (Able Scientific, Australia) on a 12 h light/dark cycle, with food and water available *ad libitum* (Westmead Hospital small animal research facility). Mice were acclimatized (d-6 to d0) prior to experiments, followed by run-in (d1-d3) in the experimental room to introduce the new gelatine food [45] (10% w/v Davis gelatine, GELITA NZ Ltd; 10% w/v Splenda artificial sweetener, Johnson-Johnson Pacific Pty Ltd, Australia; 10% v/v flavouring (Flavouring Essence Imitation Strawberry, Queen Fine Foods Pty. Ltd. QLD, Australia). Mice were fasted for 6 h, allowed access to gelatine food and then normal food was continuously available. Bacteria (1 mL culture) carrying a given plasmid were resuspended in PBS to an OD_600_ of ~0.4–0.5 and fed to mice on specified days in gelatine, with antibiotics in drinking water (20 mg/L) to follow, as previously described [45]. Mice were euthanized by an overdose of CO_2_ immediately after completion of experiments.

Group 1 received gelatine with no antibiotics. Group 2 received gelatine containing antibiotics. Groups 3, 4, 5 and 6 received bacteria with target plasmid (pEl1573 or pJIE512b, respectively) conferring cefotaxime (CTX) resistance in gelatine plus CTX to select for the plasmid. Only groups 4 and 6 subsequently received bacteria with the matching interference plasmid (TET^R^ FOS^R^) plus TET to select for it, then CTX in water at the end of the protocol to select for any residual resistant bacteria (Table 3). On the specified days each mouse was briefly transferred into a separate plastic box for weighing and to collect fresh faeces. Faeces (100 μg/mouse) was suspended in 1 mL PBS, dilutions plated on CHROMagar with appropriate antibiotics and the number of *E*. *coli*/100 μg faeces after 16 h incubation at 37°C calculated.

**Table 3:** Protocol for mouse experiments.

Day(s)	Group 1	Group 2	Group 3	Group 5	Group 4	Group 6
	Controls	Antibiotics	Antibiotics+resistance plasmid		Antibiotics+resistance+interference plasmid	
^-^6-0	acclimatization—unrestricted normal diet and water					
1–3	run-in of gelatine food protocol (6 h fast, gelatine food 3 h, unrestricted normal diet and water)					
4–6	gelatine	gelatine+CTX	gelatine+CTX+(J53Azi^r^+pEl1573)~2x10^6^ cfu/cage/day	gelatine+CTX+ (UB5201Rf+pJIE512b)~2x10^6^ cfu/cage/day	gelatine+CTX+(J53Azi^r^+pEl1573)~2x10^6^ cfu/cage/day	gelatine+CTX+ (UB5201Rf+pJIE512b)~2x10^6^ cfu/cage/day
7	unrestricted normal diet and water					
8–10	gelatine	gelatine+TET	gelatine	gelatine	gelatine+TET+(UB5201Rf+pJIMK46)~6x10^7^ cfu/cage/day	gelatine+TET+(J53Azi^r^+pJIMK56)~7x10^7^ cfu/cage/day
11	unrestricted normal diet and water					
12	move to clean cages					
13–22	unrestricted normal diet and water					
23–24	unrestricted normal diet and water				CTX in water	
28	end of experiment					

## Results

### Strong association between replicon type and addiction systems

A survey of the literature and available sequences confirmed that addiction systems are common among conjugative plasmids in the *Enterobacteriaceae*, with predictable associations between addiction systems and replicon types (Table 4). All IncL/M and IncI1 plasmids available in GenBank (November 2016) were examined with tools designed to search for putative addiction systems [46, 47]. A single addiction system (*pemIK*) was evident in 50/57 IncL/M plasmid sequences, most (n = 42/50) encoding an identical PemI antitoxin, with a single conservative amino acid change (Val to Ala at position 79) in seven and this change plus Ala80Thr in the eighth. Similarly, all 125 available IncI1 plasmid sequences had the *pndBCA* addiction system with identical *pndB* antisense RNA (antitoxin) sequences. An additional putative *relE*-RHH-like addiction system was found in 72 of these 125 IncI1 plasmids, including pJIE512b.

**Table 4:** Associated replicon types and addiction systems in *Enterobacteriaceae* plasmids.

Replicon	Associated addiction systems	Associated resistance genes	Source/Reference
IncF	*pemIK*, *ccdAB*, *hok-sok*, *vagCD*	*bla*_CTX-M_, *bla*_KPC_, *bla*_CMY-2_-like, *bla*_DHA_	[20, 21, 48, 49]
IncL/M	*pemIK*	*bla*_IMP_, *bla*_CTX-M_, *bla*_TEM_	[20, 21, 40]
IncI1	*pndBCA*, *relE-RHH*^a^ (in some)	*bla*_CMY-2_-like, *bla*_CTX-M_, *bla*_TEM_	[20–22]
IncA/C	*relE-vapI*^a^	*bla*_CMY-2_-like, *bla*_NDM_, *bla*_SHV_, *bla*_VEB_	
IncHI2	*vagCD*	*bla*_CTX-M_, *bla*_SHV_	[21]
IncN	*stbBC*	*bla*_KPC_, *bla*_CTX-M_, *bla*_SHV_	[50, 51]
IncX	*hicA-hicB-*like (in some)	*bla*_CTX-M_, *bla*_KPC_, *bla*_NDM_	[52–55]

^*a*^ putative systems identified using RASTA-Bacteria (http://genoweb1.irisa.fr/duals/RASTA-Bacteria/) and TA-finder (http://202.120.12.133/TAfinder/TAfinder.php).

### *In vitro* cure of antibiotic resistance plasmids from *Enterobacteriaceae*

The conjugative plasmids pEl1573 [40] and pJIBE401 [56] are almost identical (one nucleotide difference in the MRR) representatives of an IncL/M plasmid type that is common in Sydney hospitals [4, 39]. They carry genes encoding resistance to gentamicin (GEN) in addition to *bla*_IMP-4_ encoding a metallo-β-lactamase that efficiently hydrolyses extended spectrum β-lactams (e.g. cefotaxime, CTX) and carbapenems.

In order to evaluate plasmid stability, *E*. *coli* UB5201Rf carrying only pEl1573 [56] was passaged in serial culture in LB without antibiotic selection for 100 consecutive days. All of 100 colonies retrieved on antibiotic-free growth media at the end of this period were resistant to GEN and CTX on subculture and all were still positive by PCR with primers specific for *bla*_IMP-4_ (CTX^R^) and the IncL/M plasmid replicon (S1 Table), confirming the long-term stability of pEl1573 in the absence of antibiotic selection.

By contrast, provision of the specific antitoxin gene *pemI* from pEl1573 expressed *in trans* from the unrepressed P*lac* promoter in a high-copy chloramphenicol-resistant (CHL^R^) vector (pJIMK3; Table 1) transformed into the same strain (*E*. *coli* UB5201Rf with pEl1573) resulted in significant loss of pEl1573. After six passages over 48 h in LB supplemented with CHL, ~30% of *E*. *coli* colonies recovered were GEN^S^ CTX^S^ and no longer yielded *bla*_IMP-4_ or IncL/M amplicons with specific PCR. The remainder retained the antibiotic-resistant phenotype and genetic markers of pEl1573.

The pEl1573 IncL/M replicon region (*rep*) was then added to pJIMK3 to generate pJIMK25, in which *pemI* and IncL/M *rep* are constitutively expressed from P*lac* (Table 1), which was transformed into *E*. *coli* UB5201Rf with pEl1573. After overnight incubation in LB supplemented with CHL, as purifying selection for pJIMK25, all transformants were GEN^S^ CTX^S^ and *bla*_IMP-4_ was no longer detected by specific PCR. Complete loss of pEl1573 was confirmed by gel electrophoresis after S1 nuclease treatment of extracted DNA to linearise plasmids [57] (not shown). Complete loss of pEl1573 was also observed after specific expression of IncL/M *rep* and *pemI* from an arabinose-inducible promoter in a low-copy vector (pJIMK39) in *E*. *coli* UB5201Rf with pEl1573 (Table 2) after 6 h growth in the presence of arabinose (expressing IncL/M *rep* and *pemI*). By contrast, pEl1573 and pJIMK39 stably coexisted in the presence of glucose (the promoter-repressed state; data not shown), confirming that the effect was wholly attributable to expression of specific *rep* and antitoxin.

### Construction of conjugative interference plasmids

Having demonstrated plasmid displacement using small high copy number plasmids, specific conjugative ‘interference plasmids’ were constructed. The resistance region and principal toxin gene of pJIBE401 and pJIE512b were replaced with *tetA* or *fosA3*, retaining the antitoxin gene and introducing resistance to tetracycline (TET^R^) and fosfomycin (FOS^R^) (Fig 2). Sequencing of these specific interference plasmids (pJIMK46, pJIMK56) confirmed that each was otherwise identical to their parent resistance plasmid (pEl1573/pJIBE401 and pJIE512b, respectively).

In order to gauge the likely impact of exclusion systems and the need for purifying selection in favour of interference plasmids, the recovery of interference plasmids after prolonged (overnight) *in vitro* filter mating was examined. Establishment of both IncI1 and IncL/M interference plasmids was around 8-fold less efficient when incubated with cells in which the incompatible resistance plasmid was already resident (Table 5).

**Table 5:** Effect of entry exclusion on conjugative transfer efficiency.

Donor	Recipient	Donor(cfu/ml)	Transconjugant(cfu/ml)	Conjugation frequency(transconjugants/donor)	Difference in frequency(fold)
UB5201Rf (pJIMK46); RIF^R^-TET^R^	J53Azi; AZI^R^	3.6x10^8^	6.3 x10^7^(AZI^R^-TET^R^)	1.75 x10^-1^	8.02
UB5201Rf (pJIMK46); RIF^R^-TET^R^	J53Azi(pEl1573); AZI^R^-CTX^R^	3.6x10^8^	7.86x10^6^ (AZI^R^-TET^R^)	2.18 x10^-2^	
J53Azi (pJIMK56);AZI^R^-TET^R^	UB5201Rf; RIF^R^	5.6x10^7^	1.9x10^6^(RIF^R^-TET^R^)	3.39 x10^-2^	7.9
J53Azi (pJIMK56);AZI^R^-TET^R^	UB5201Rf (pJIE512b);RIF^R^-CTX^R^	5.6x10^7^	2.4x10^5^(RIF^R^-TET^R^)	4.28 x10^-3^	

**Fig 3:**
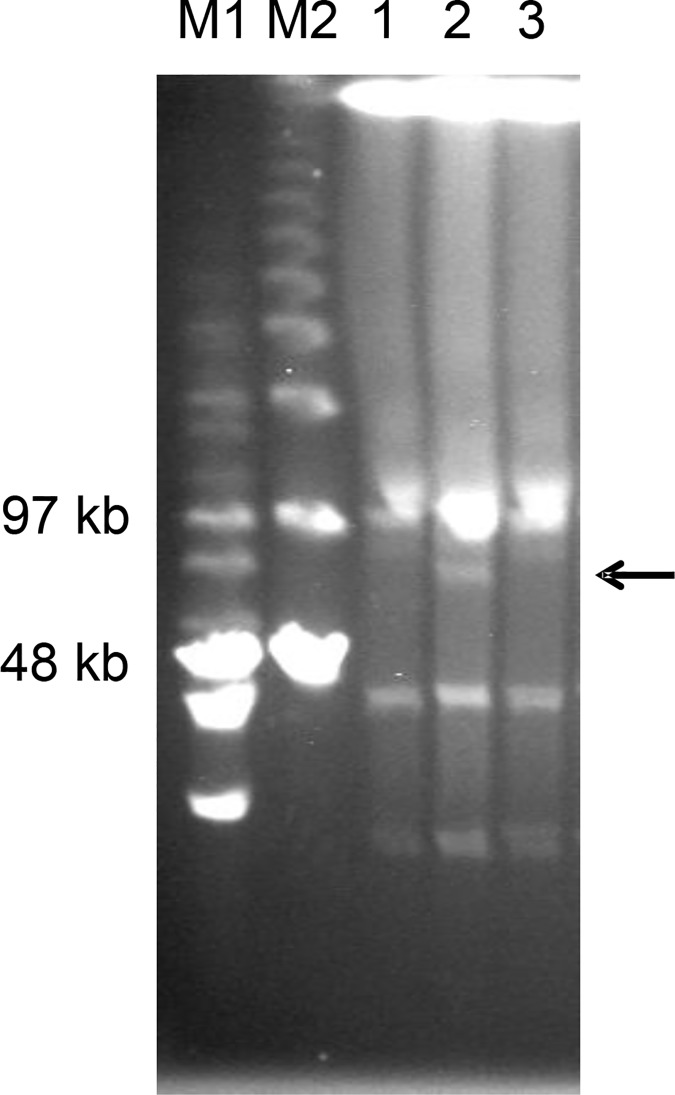
Acquisition and loss of pEl1573 from *K*. *pneumoniae* 13883. Pulsed-field gel electrophoresis of S1-endonuclease treated extracts of Kp13883 before (1) and after (2) acquisition of pEl1573 (horizontal arrow) and after cure (3), showing other ‘bystander’ plasmids. M1, Mid-range and M2, Lambda PFG ladders (New England Biolabs, USA).

Conjugation of the interference plasmid pJIMK46 (TET^R^) into rifampicin-resistant (RIF^R^) *E*. *coli*, *K*. *pneumoniae*, *Citrobacter freundii* or *Morganella morganii* (Table 2) carrying the respective CTX^R^ resistance plasmid (pEl1573 or pJIE512b) appeared to result in loss of the resistance plasmid only after purifying selection on RIF-TET agar, with bystander plasmids preserved (Fig 3). Plasmid curing was confirmed by PCR for *bla*_IMP-4_ (pEl1573) or *bla*_CMY-2_ (pJIE512b). No CTX^R^ (resistance) or TET^R^ (interference) bacteria could be detected subsequently by selective subculture.

## *In vivo* cure of AbR plasmids from mouse gut

Having demonstrated *in vitro* efficacy and specificity of interference plasmids, BALB/c mice were selected as a suitable model for *in vivo* study [58]. Groups of three mice per cage were shown to be initially free of CTX^R^ *Enterobacteriaceae* by faecal culture for three consecutive days. Protocols based on published work [45] were used to introduce resistance and then interference plasmids in bacteria with different chromosomal resistance markers (rifampicin (RIF^R^) or azide (AZI^R^; Table 3), to allow tracking of both strains and plasmids by differential subculture. Mouse weights were stable throughout and no feeding disturbance or diarrhoea was observed during the 28 day protocols (not shown).

In a preliminary experiment, mice received pEl1573 (TET^S^ CTX^R^) in RIF^R^ *E*. *coli* and then pJIMK46 (TET^R^ CTX^S^) in AZI^R^ *E*. *coli*, the latter being administered either with or without TET. In those mice that received pJIMK46 in AZI^R^ *E*. *coli*, pJIMK46 was found in all culturable *E*. *coli* after three days of purifying TET selection while pEl1573 was no longer detected. In those mice that received pJIMK46 without purifying TET selection, the RIF^R^ *E*. *coli* originally used to introduce pEl1573 were found to contain both pEl1573 and pJIMK46 in an approximate ~10:1 ratio after three days. This relatively poor penetration of pJIMK46 into the RIF^R^ *E*. *coli* population in the absence of TET selection is consistent with exclusion by the resident incompatible pEl1573 and confirms the need for purifying selection *in vivo*.

A more detailed experiment introducing either pEl1573 (IncL/M; CTX^R^) or pJIE512b (IncI1; CTX^R^) and then the respective interference plasmid (pJIMK46/pJIMK56; TET^R^) was next conducted. Host *E*. *coli* strains used to introduce pJIE512b (AZI^R^) or pJIMK56 (RIF^R^) were switched for pEl1573/pJIMK46 to ensure that each *E*. *coli* strain was used both as initial colonizer and also to introduce interference plasmid, to control for any strain-related effects as experimental confounders.

**Fig 4:**
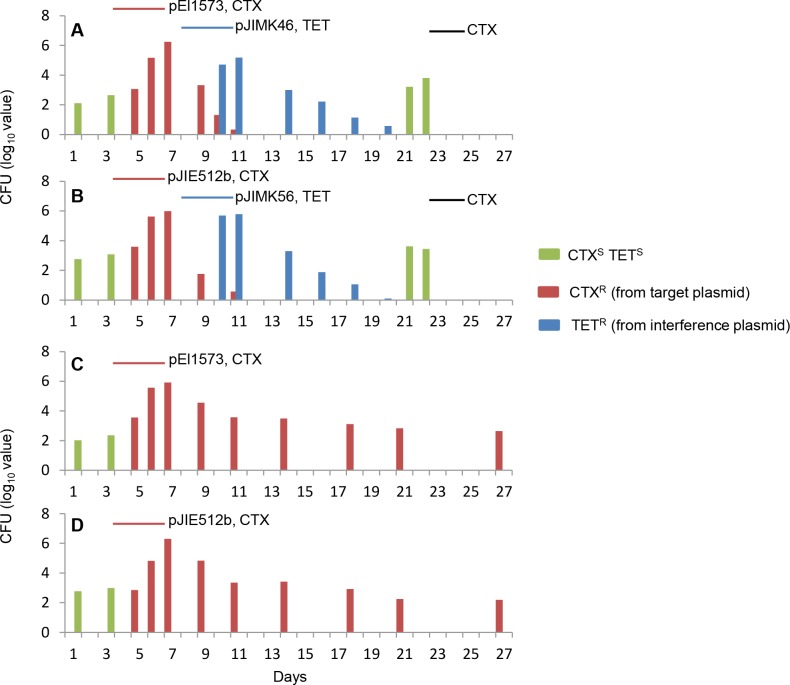
*In vivo* cure of antibiotic resistance plasmids. CTX^S^TET^S^ *E*. *coli* (green) were detected in all four groups of mice at the start of the experiments (**A**-**D**). All groups of mice were then fed bacteria carrying CTX^R^ target plasmid (pEl1573 or pJIE512b) with CTX, days 4–6 (red lines, **A**-**D**) and CTX^R^ *E*. *coli* (red) appeared. Two groups of mice (**A**, **B**) then received the corresponding TET^R^ interference plasmid (pJIMK46 or pJIMK56) with TET, days 8–10 (blue lines) resulting in a decline in number of CTX^R^ *E*. *coli*. TET^R^ *E*. *coli* (blue) also appeared and then declined. CTX^S^TET^S^ *E*. *coli* appeared again after curing of target and interference plasmids but were killed by CTX administered on days 23–24 (black lines, **A**, **B**). CTX^R^ *E*. *coli* persisted to end of protocol in control groups that did not receive interference plasmid (**C**, **D**).

Different CTX^R^ *E*. *coli* populations were retrieved from faeces immediately after introduction of pEl1573 or pJIE512b resistance plasmid, indicating effective colonization of *E*. *coli* already resident in the mouse gut in the presence of CTX selection (Tables 6 and 7). In those groups of mice that then received specific interference plasmid with TET selection, TET^R^ *E*. *coli* with either RIF^R^ or AZI^R^ chromosomal markers (used to bring in resistance or interference plasmid) or with no such markers (previously resident) were soon detected in approximately equal proportions (Fig 4A and 4B; Tables 6 and 7). After the three days of administration of interference plasmid (TET^R^) along with relevant antibiotic (TET) in water, CTX^R^ *Enterobacteriaceae* were no longer culturable. None of 360 colonies of TET^R^ *E*. *coli* (AZI^R^-TET^R^ for curing pEl1573 and RIF^R^-TET^R^ for curing pJIE512b) subcultured from faeces in each experiment were CTX^R^ and CTX^R^ genes (*bla*_IMP-4_ or *bla*_CMY-2_) could not be amplified from cultured bacteria or faecal extracts. This indicated that no interference plasmids had acquired CTX^R^, nor had resistance plasmids acquired TET^R^, nor did CTX^R^ and TET^R^ traits persist together in any isolate.

**Table 6:** *In vivo* cure of pEl1573-colonized mice.

Day	Mouse no.	Group 3 (resistance plasmid)J53Azi^r^+pEl1573 D4-6		Group 4 (resistance plasmid followed by interference plasmid)J35Azi^r^+pEl1573 D4-6; UB5201Rf+pJIMK46 D8-10; CTX D23-24					
		CTX^R^	AZI^R^-CTX^R^	CTX^R^	AZI^R^-CTX^R^	TET^R^	RIF^R^-TET^R^	AZI^R^-TET^R^	CTX^S^
D5	M1	1.7x10^3^	1.5x10^3^	2.3x10^3^	1.9x10^3^				
	M2	2.1x10^4^	1.9x10^4^	3.5x10^3^	2.9x10^3^				
	M3	1.3x10^3^	1.0x10^3^	1.9x10^2^	1.5x10^2^				
D6	M1	2.7x10^5^	2.1x10^5^	1.8x10^5^	1.5x10^5^				
	M2	3.4x10^5^	2.2x10^5^	3.6x10^5^	3.1x10^5^				
	M3	5.4x10^5^	4.8x10^5^	4.7x10^4^	4.0x10^4^				
D7	M1	3.4x10^5^	2.8x10^5^	3.4x10^7^	2.9x10^7^				
	M2	3.7x10^6^	3.2x10^6^	3.7x10^5^	3.5x10^5^				
	M3	4.3x10^5^	3.8x10^5^	4.3x10^5^	4.1x10^5^				
D9	M1	5.6x10^4^		3.1x 10^3^			3.6x10^4^		
	M2	3.2x10^4^		6.2x10^2^			4.3x10^4^		
	M3	2.5x10^4^		4.7x10^3^			5.4x10^4^		
D10	M1			nil		2.5x10^5^	5.4x10^4^	3.6x10^5^	
	M2			4.0x10^1^		2.9x10^3^	2.1x10^3^	3.8x10^2^	
	M3			2.1x 10^2^		1.9x10^5^	1.5x10^4^	4.8x10^4^	
D11	M1	1.8x10^4^		nil		3.0x10^5^	4.8x10^4^	5.3x10^4^	
	M2	2.3x10^3^		1.0x10^1^		1.0x10^5^	1.2x10^4^	8.9x10^3^	
	M3	1.3x10^3^		nil		1.2x10^5^	3.1x10^4^	3.5x10^4^	
D12	M1			nil				1.4x10^4^	
	M2			nil				3.2x10^4^	
	M3			nil				1.8x10^4^	
D14	M1	5.1x10^3^		nil		1.1x10^3^			
	M2	3.1x10^3^		nil		2.0x10^2^			
	M3	1.8x10^3^		nil		4.3x10^3^			
D16	M1			nil		3.0x10^2^			
	M2			nil		1.3x10^2^			
	M3			nil		1.1x10^2^			
D18	M1	3.6x10^3^		nil		1.3x10^2^			
	M2	8.8x10^2^		nil		nil			
	M3	6.7x10^2^		nil		2.0x10^1^			
D20	M1			nil		5.0x10^1^			
	M2			nil		nil			
	M3			nil		nil			
D21	M1	7.2x10^2^		nil		nil			2.4x10^3^
	M2	9.3x10^2^		nil		nil			5.6x10^2^
	M3	4.7x10^2^		nil		nil			3.1x10^3^
D22	M1			nil		nil			8.3x10^3^
	M2			nil		nil			7.2x10^3^
	M3			nil		nil			4.3x10^3^
D27	M1	4.4x10^2^		nil					nil
	M2	3.9x10^2^		nil					nil
	M3	5.0x10^2^		nil					nil

*E*. *coli*/100 μg faeces with indicated antibiotic resistance/susceptibility phenotype. CTX, cefotaxime; AZI, azide; TET, tetracycline; RIF, rifampicin. Mice were transferred to clean cages D12 and CTX re-administered D23-24 (Group 4 only). Blank, not applicable/not determined; nil, none cultured.

**Table 7:** *In vivo* cure of pJIE512b-colonized mice.

Day	Mouse no.	Group 5 (resistance plasmid)UB5201+pJIE512b D4-6		Group 6 (resistance plasmid followed by interference plasmid)UB5201+pJIE512b D4-6; J35Azi^r^+pJIMK56 D8-10; CTX D23-24					
		CTX^R^	RIF^R^-CTX^R^	CTX^R^	RIF^R^-CTX^R^	TET^R^	RIF^R^-TET^R^	AZI^R^-TET^R^	CTX^S^
D5	M1	2.5x10^2^	2.1x10^2^	3.1x10^3^	3.0x10^3^				
	M2	1.9x10^2^	1.5x10^2^	1.1x10^4^	9.5x10^3^				
	M3	7.3x10^3^	7.0x10^3^	1.8x10^3^	1.3x10^3^				
D6	M1	7.1x10^4^	6.4x10^4^	5.2x10^5^	5.0x10^5^				
	M2	1.6x10^5^	1.5x10^5^	3.9x10^5^	3.2x10^5^				
	M3	2.4x10^4^	2.2x10^4^	3.5x10^5^	3.1x10^5^				
D7	M1	1.1x10^6^	1.0x10^6^	3.1x10^7^	3.1x10^7^				
	M2	6.1x10^5^	5.7x10^5^	1.3x10^5^	1.2x10^5^				
	M3	1.2x10^7^	1.0x10^7^	2.2x10^5^	2.0x10^5^				
D9	M1	1.1x10^5^		5.1x10^2^				1.4X10^3^	
	M2	6.5x10^4^		3.7x10^2^				3.9x10^4^	
	M3	4.3x10^4^		1.3x10^3^				5.3x10^4^	
D10	M1			2.0x10^2^		8.3x10^5^	1.7x10^3^	1.8x10^4^	
	M2			4.0x10^1^		3.3x10^5^	2.3x10^4^	1.5x10^4^	
	M3			nil		4.2x10^5^	1.9x10^4^	3.1x10^3^	
D11	M1	1.3x10^3^		5.0x10^1^		8.4x10^5^	1.6x10^4^	1.4x10^5^	
	M2	3.3x10^3^		nil		3.8x10^5^	1.9x10^4^	3.1x10^5^	
	M3	2.5x10^3^		nil		7.2x10^5^	3.0x10^4^	8.7x10^4^	
D12	M1			nil			3.2x10^3^		
	M2			nil			2.0x10^4^		
	M3			nil			8.6x10^3^		
D14	M1	1.1x10^3^		nil		2.5x10^3^			
	M2	5.1x10^3^		nil		1.5x10^3^			
	M3	3.1x10^3^		nil		2.0x10^3^			
D16	M1			nil		1.1x10^2^			
	M2			nil		1.0x10^2^			
	M3			nil		4.0x10^1^			
D18	M1	3.6x10^2^		nil		nil			
	M2	7.2x10^2^		nil		3.0x10^1^			
	M3	2.1x10^3^		nil		5.0x10^1^			
D20	M1			nil		nil			
	M2			nil		nil			
	M3			nil		2.0x10^1^			
D21	M1	2.3x10^2^		nil		nil			5.3x10^3^
	M2	1.2x10^2^		nil		nil			6.4x10^3^
	M3	2.0x10^2^		nil		nil			2.1x10^3^
D22	M1			nil		nil			5.5x10^3^
	M2			nil		nil			8.3x10^2^
	M3			nil		nil			4.6x10^3^
D27	M1	1.0x10^2^		nil					nil
	M2	2.2x10^2^		nil					nil
	M3	1.6x10^2^		nil					nil

*E*. *coli/*100 μg faeces with indicated antibiotic resistance/susceptibility phenotype. CTX, cefotaxime; AZI, azide; TET, tetracycline; RIF, rifampicin. Mice were transferred to clean cages D12 and CTX re-administered D23-24 (Group 6 only). Blank, not applicable/not determined; nil, none cultured.

After administration of interference plasmid (TET^R^) to mice in the treatment groups, all mice were transferred into new cages to exclude reinfection from residual faecal contamination of their environment. Ten days after cessation of TET, no TET^R^ *Enterobacteriaceae* could be cultured from mice who had received TET^R^ interference plasmid (Fig 4A and 4B; Tables 6 and 7). None of the specific resistance (*bla*_CMY-2_, *bla*_IMP-4_, *tetA*) or replicon (IncL/M and IncI1 *rep*) genes from any of the introduced plasmids could be amplified from faecal pellets, confirming the loss of the interference plasmid (which lacks the addiction toxin gene). Only antibiotic-susceptible (originally resident, TET^S^ CTX^S^) *E*. *coli*, AZI^R^ TET^S^ CTX^S^ *E*. *coli* and RIF^R^ TET^S^ CTX^S^ *E*. *coli* (bacteria used to introduce resistance or interference plasmid, now carrying neither) were retrieved, and these were present in comparable proportions (~10^4^−10^5^ cfu/mg of faeces; Tables 6 and 7).

CTX was finally re-administered (days 23, 24) to select for any residual resistance CTX^R^ plasmids or transferred genes that may not have been detected by culture or direct PCR. *bla*_IMP-4_ and *bla*_CMY-2_ (CTX^R^) remained undetectable in stool extracts, and neither CTX^R^ or TET^R^ bacteria could be recovered (Fig 4, Tables 6 and 7). Elimination of CTX^S^ *E*. *coli* populations further demonstrated the return of the efficacy of the antibiotic that had been rendered ineffective by the presence of the resistance plasmids.

In control mice that received the resistance plasmid but not the interference plasmid, CTX^R^ bacteria remained (Fig 4C and 4D; Tables 6 and 7) and specific *rep* and resistance genes remained readily detectable throughout the experiment, confirming the addictive nature of the resistance plasmids.

## Discussion

Eradication of resistant bacterial populations by more powerful antibiotics continues the escalation of the antibiotic arms race, leaves the microflora open to invasion by other species, and will not save antibiotics trusted for decades. This study shows that addictive antibiotic resistance plasmids can be specifically and completely eradicated from enteric bacterial populations and these bacterial populations recovered in their antibiotic-susceptible state *in vivo*. Effective targeting of incompatibility and addiction may provide solutions for a variety of antibiotic resistance plasmids, and high levels of conservation in these systems may even allow specific off-the-shelf solutions to be developed in the future. Two representative antibiotic resistance plasmids with different replicon and entry exclusion systems and with the two most common types of addiction systems were used to demonstrate this. IncL/M and IncI1 plasmids are found in different species of *Enterobacteriaceae*, often carry genes conferring resistance to important β-lactam and/or to aminoglycoside antibiotics, and are typical examples of plasmids that require specific eradication.

The ability to generalize this approach to other replicons and addiction systems must now be systematically tested: addiction systems are yet to be characterized, some may interact, and not all apparently similar systems are interchangeable. PemI and PemK encoded by IncL/M plasmids such as pEl1573, for example, differ by 4/84 amino acids and 10/133 amino acids respectively from PemI and PemK encoded by IncF plasmids, in which they appear to function in conjunction with the *hok-sok* addiction system [59]. Further, while the putative *relE*-RHH-like addiction system identified in pJIE512b clearly does not prevent plasmid loss from mouse gut microflora *in vivo* and neutralization of *pndBCA* alone was sufficient to cure this plasmid, it is not known whether this would be true for microflora containing different host species than those in the mouse gut model used here. Evidently this system is not essential for IncI1 plasmid stability in all *E*. *coli* populations in which it is found.

The results regarding the inhibitory effect of entry exclusion are at the lowest end of published data [13] but are internally consistent both *in vitro* and *in vivo* and in both systems. Entry exclusion of the IncI1 plasmid R64, which is closely related to the target plasmid pJIE512b and derivative pJIMK56, has been reported to inhibit *in vitro* conjugative transfer by ~700-fold in 90 min surface mating studies [14]. The differences reported here may relate to differences between pJIE512b (GenBank accession no. HG970648) and R64 (AP005147) in both *excA* and the adjacent *traY* (13/220 and 91/744 amino acids, respectively), as these differences are in key functional regions of the proteins [14] (S1 Fig). Strains expressing standard PSK/addiction system toxins may also have a relative fitness disadvantage *in vivo* and in a longer mating protocols such as used here, when compared to toxin-deleted interference plasmids, although this was not specifically tested here.

**Fig 5:**
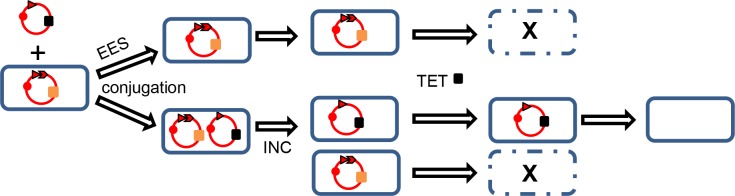
Exclusion and incompatibility. Replicon (solid circle), antitoxin and toxin genes (arrowhead, arrow) and antibiotic resistance genes (CTX^R^, orange and TET^R^, black solid blocks). Interference plasmid not excluded by entry exclusion system (EES) is incompatible (INC) with resident CTX^R^ plasmid and is selected by TET.

In the system used here, TET (or FOS) resistance can be used as purifying selection for bacteria which acquire and retain the interference plasmid at the expense of the incompatible CTX^R^ target plasmid. Cells from which the TET^R^ interference plasmid is lost, or that it does not enter at all (e.g. due to entry exclusion), are killed by TET (Fig 5). With the subsequent loss of the non-addictive interference plasmid in the absence of specific selection, all cells are thus free of both the interference plasmid and the original target resistance plasmid.

This process should therefore be generally efficacious even at low plasmid transfer efficiencies in the presence of brief positive selection for the interference plasmid, but there are potential risks to be considered. Theoretically, homologous recombination might restore toxin or antibiotic resistance genes to an interference plasmid, although a less resistant addictive plasmid or a non-addictive resistance plasmid both seem preferable options to persistence of the original addictive resistance plasmid. Nevertheless, no evidence of recombination was found in any experiments here. This is not unexpected as large co-integrates of interference and resistance plasmids would be subject to multimer resolution mechanisms and to the relative plasmid instability that evidently arises from specific antitoxin excess. Simultaneous antibiotic selection for both (mutually incompatible) plasmids might also favour chromosomal acquisition of resistance genes from the interference plasmid. The associated genetic elements that usually mobilize these genes were deliberately removed and transfer of *fosA3* or *tetA* was not observed.

It is also theoretically possible that strong and simultaneous co-selection for both target and interference plasmids might select for a mutation in the replication region that restores compatibility. However, replicon diversity is naturally limited by strong functional constraints. Even closely evolutionarily related compatible plasmids with cross-complementing primases and helicases have highly specific *oriV*-Rep DNA binding requirements [60]. Even if target plasmids develop *oriV* mutations so that they are no longer incompatible and can then co-inhabit a cell with the interference plasmid, these plasmids are still expected to be rendered relatively unstable by specific antitoxin excess. It is also noteworthy that not all plasmid T/A systems are simple addiction mechanisms nor will they necessarily behave identically in different hosts or conditions. Likewise, simple antitoxin excess, evidently effective in these experiments, may also be insufficient in some systems.

Purifying selection using antibiotics is not ideal, both because of the effects of the drug on other cells and because effective antibiotics for this purpose may be increasingly hard to identify in future. TET^R^ bacteria are not uncommon in the human gut but TET^R^ (or FOS^R^) populations that arise by plasmid acquisition will not only lose their TET^R^ or FOS^R^ plasmids spontaneously but are made immediately vulnerable to other commonly used antibiotics (e.g. GEN, CTX) in the process. A target plasmid that acquires FOS^R^ by recombination with the interference plasmid will likely acquire the immediately adjacent antitoxin (see Fig 2). An important future challenge is to develop interference plasmids with non-antibiotic selection but the inclusion of *fosA3* also means that plasmid eradication is compatible with existing use of fosfomycin as a ‘rescue’ therapy of last resort [61]. A final caveat is that not all antibiotic resistance is carried on large plasmids that can be manipulated in this way. Nearly half of all severe sepsis is due to Gram-positive bacteria such as *Staphylococci* and *Streptococci*, in which much of the important resistance is chromosomally encoded, as it is in many clinically important Gram-negative bacteria such as *Pseudomonas* and *Acinetobacter*.

Specific plasmid interference is not only a useful research tool but may be suitable for clinical use in colonized individuals. The ability to eradicate plasmids detected in the gut flora of patients may prevent later development of antibiotic resistant sepsis, a condition with potentially lethal consequences [5]. Hospitals managing critically ill people at high risk of infection now routinely screen for antibiotic resistance using genetic methods and could similarly provide rapid identification of specific plasmid markers. The simple oral administration route makes *in vivo* plasmid curing approaches highly feasible, and may allow us to protect important medical advances from the growing threat of antibiotic resistance.

## Supporting information

S1 Table Primers used in this study. (DOCX)

S1 Fig Comparison of TraY and Exc amino acid sequences of R64 and pJIE512b. The internal variable region of TraY (**A,** aa 430–522) and C-terminal region of Exc (**B**) are shown. Variable amino acids are shown by black shading, numbers correspond to amino acid positions in proteins.
(DOCX)
