# Supplementary material for: Plasmid interference for curing antibiotic resistance plasmids in vivo
Source: PLoS One. 2017 Feb 28;12(2):e0172913. doi: 10.1371/journal.pone.0172913 (PMC5330492; doi:10.1371/journal.pone.0172913)
Supplement: S1 Table — (DOCX) [file pone.0172913.s001.docx]

**S1Table.** Primers used in this study

| Primer | Sequence (5′-3′)^a^ | Comment | GenBank  Reference |  |
| --- | --- | --- | --- | --- |
| PemI-F1 | TTTGGCTGAAAGTTATATTTTTATT | to amplify pEl1573 *pemI* with native promoter | JX101693 | |
| PemI-R1 | TGCGGTAGGATCAAGCGAGA |  |  | |
| IncL/M-F | GCCGAATATGGCGGGTTTTT | to amplify pEl1573 L/M replication genes (*repC*+*repB*+*repA*) | JX101693 | |
| IncL/M-R | AGGCGGCGGTAAGAGAATTACAG |  |  | |
| FosA3-F2 | ATAGAGCGGGGATTAGTGTGG | to amplify *fosA3* gene with native promoter | JF411006 | |
| FosA3-R1 | GCTGTGGATCTGCACGTTGAA |  |  | |
| TetA-F | GAGCCTCTACGCCGACCTCA | to amplify *tetA* gene of plasmid N3 with native promoter | FR850039 | |
| TetA-R | GTAGCCGGAAGTCGCCTTGA |  |  | |
| U-pemK-F | TACATCAGCTGCCCGGATCA | in pEl1573 *tir* gene, upstream of *pemK* | JX101693 | |
| U-pemK-R | **CCACACTAATCCCCGCTCTAT**AGACGGGTCACGCGGTTAAA | in pEl1573 *pemK* gene |  | |
| D-pemK-F | **TTCAACGTGCAGATCCACAGC**CCCATTGTTTCGACAGAGAACC | in non-coding region, downstream of *pemK* | JX101693 | |
| D-pemK-R | GCGGGCATCACGCATAGAAT | in *mucA* gene, downstream of *pemK* |  | |
| U-MRR-F | CGCGACCTGCTCAAATTTCC | in non-coding region, upstream of MRR | JX101693 | |
| U-MRR-R | TGAGGTCGGCGTAGAGGCTCCCCTGTTTAACGCGCCTGAG | in non-coding region, upstream of MRR |  | |
| D-MRR-F1 | TCAAGGCGACTTCCGGCTACTTTTGTTCAGGTGCGGAGCCAGG | in hypothetical gene, downstream of MRR | JX101693 | |
| D-MRR-R1 | GAAACCGTCGGCCAGGATTA | in *trbC* gene, downstream of MRR |  | |
| U-pndA-F | TTACCGGTCATGGCAAGCAG | in pJIE512b *trbA* gene, upstream of *pndA* | HG970648 | |
| U-pndA-R | TGAGGTCGGCGTAGAGGCTCTTAACTTCGTAGGCTAACGTTGCCACAA | at the end of *pndC*, overlapping with *pndA* |  | |
| D-pndA-F | TCAAGGCGACTTCCGGCTACAGCTCCAGCCGAATGCCTTT | in hypothetical gene, downstream of *pndA* | HG970648 | |
| D-pndA-R | CCGATGGATATCACGGCAGA | in non-coding region, downstream of *pndA* |  | |
| U-CMY2-F | TTGCGGAGCAGAACCTGATG | in pJIE512b *yacB* gene, upstream of *bla*_CMY-2_ | HG970648 | |
| U-CMY2-R | **CCACACTAATCCCCGCTCTAT**TGCGCCCCGATGGCGACCGTG | in pJIE512b *yacC* gene, upstream of *bla*_CMY-2_ |  | |
| D-CMY2-F | **TTCAACGTGCAGATCCACAGC**AAATCCCATGCGCATTTTGC | in non-coding region, downstream of *bla*_CMY-2_ | HG970648 | |
| D-CMY2-R | TGAGCCTTGGGCTGATCGTT | in pJIE512b *sugE* gene, downstream of *bla*_CMY-2_ |  | |
| IMP4F | atgttctagAGCAGCAACGATGT^b^ | *bla*_IMP-4_ detection | JX101693 | |
| IMP4R2 | gttctagatttagttgcttagttttgatggttt^b^ |  |  | |
| CMY-2F | CAACACGGTGCAAATCAAAC | *bla*_CMY-2_ detection | [[1](#_ENREF_1)] | |
| CMY-2R | AAAGGAGGCCCAATATCCTG |  | HG970648 | |
| L/M FW | GGATGAAAACTATCAGCATCTGAAG | L/M *rep* detection | [[2](#_ENREF_2)] | |
| L/M RV | CTGCAGGGGCGATTCTTTAGG |  |  | |
| I1 FW | CGAAAGCCGGACGGCAGAA | I1 *rep* detection | [[2](#_ENREF_2)] | |
| I1 RV | TCGTCGTTCCGCCAAGTTCGT |  |  | |

^a^Bold indicates nucleotides overlapping with *fosA3* primers; underlined indicates nucleotides overlapping with *tetA* primers

^b^Lowercase indicates additional nucleotides with *Xba*I restriction site designed for cloning *bla*_IMP-4_ in another study.

**References**

1. Song W, Lee H, Lee K, Jeong SH, Bae IK, Kim JS, et al. CTX-M-14 and CTX-M-15 enzymes are the dominant type of extended-spectrum β-lactamase in clinical isolates of *Escherichia coli* from Korea. J Med Microbiol. 2009; 58(Pt 2):261-266. doi: 10.1099/jmm.0.004507-0 PubMed PMID: 19141747.

2. Carattoli A, Bertini A, Villa L, Falbo V, Hopkins KL, Threlfall EJ. Identification of plasmids by PCR-based replicon typing. J Microbiol Methods. 2005; 63(3):219-228. doi: 10.1016/j.mimet.2005.03.018 PubMed PMID: 15935499.
