# Supplementary material for: Plasmid interference for curing antibiotic resistance plasmids in vivo
Source: PLoS One. 2017 Feb 28;12(2):e0172913. doi: 10.1371/journal.pone.0172913 (PMC5330492; doi:10.1371/journal.pone.0172913)
Supplement: S1 Fig — The internal variable region of TraY (A, aa 430–522) and C-terminal region of Exc (B) are shown. Variable amino acids are shown by black shading, numbers correspond to amino acid positions in proteins. (DOCX) [file pone.0172913.s002.docx]

**S1 Fig. Comparison of TraY and Exc amino acid sequences of R64 and pJIE512b.**

The internal variable region of TraY (**A,** aa 430-522) and C-terminal region of Exc (**B**) are shown. Variable amino acids are shown by black shading, numbers correspond to amino acid positions in proteins.


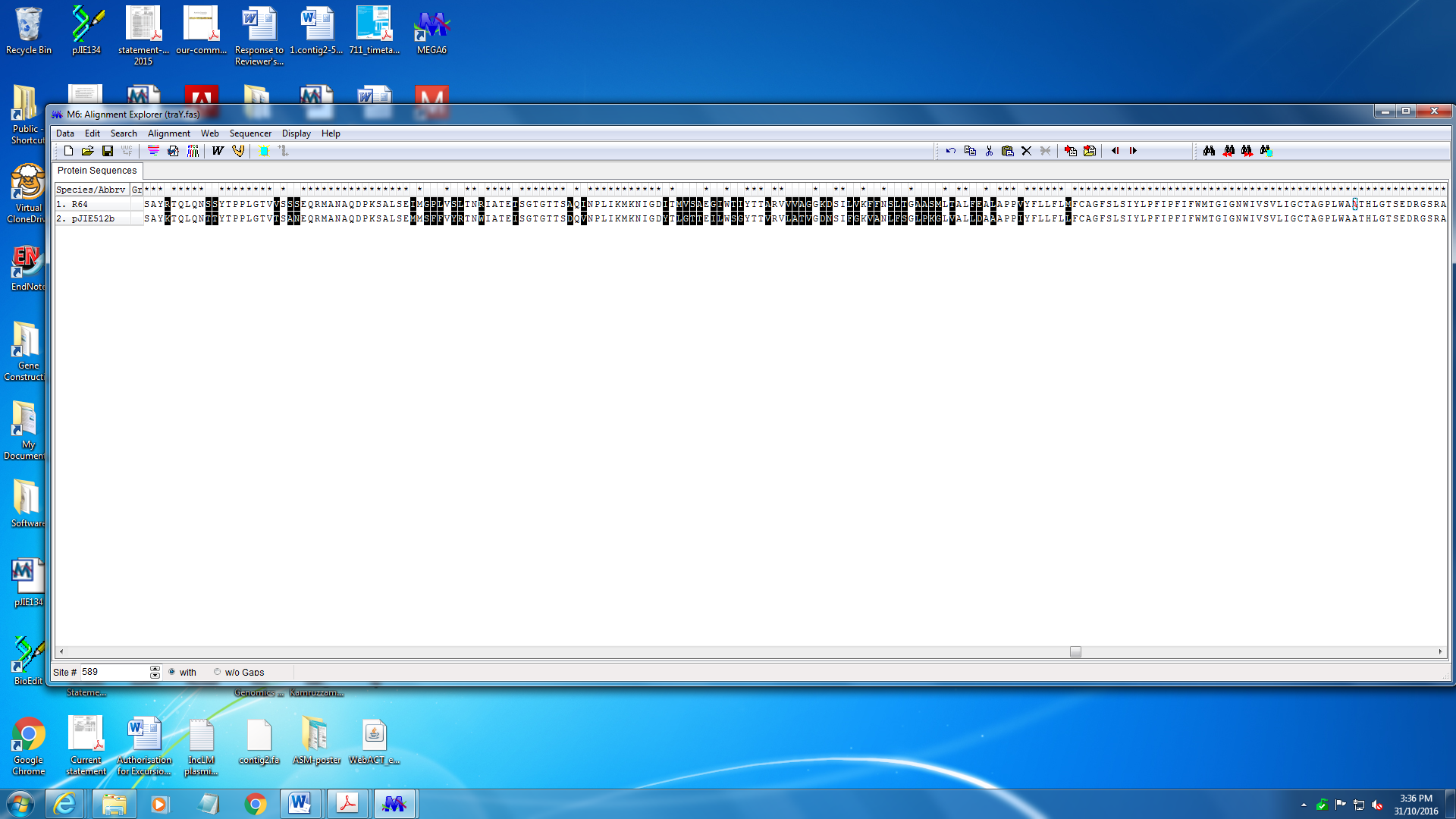

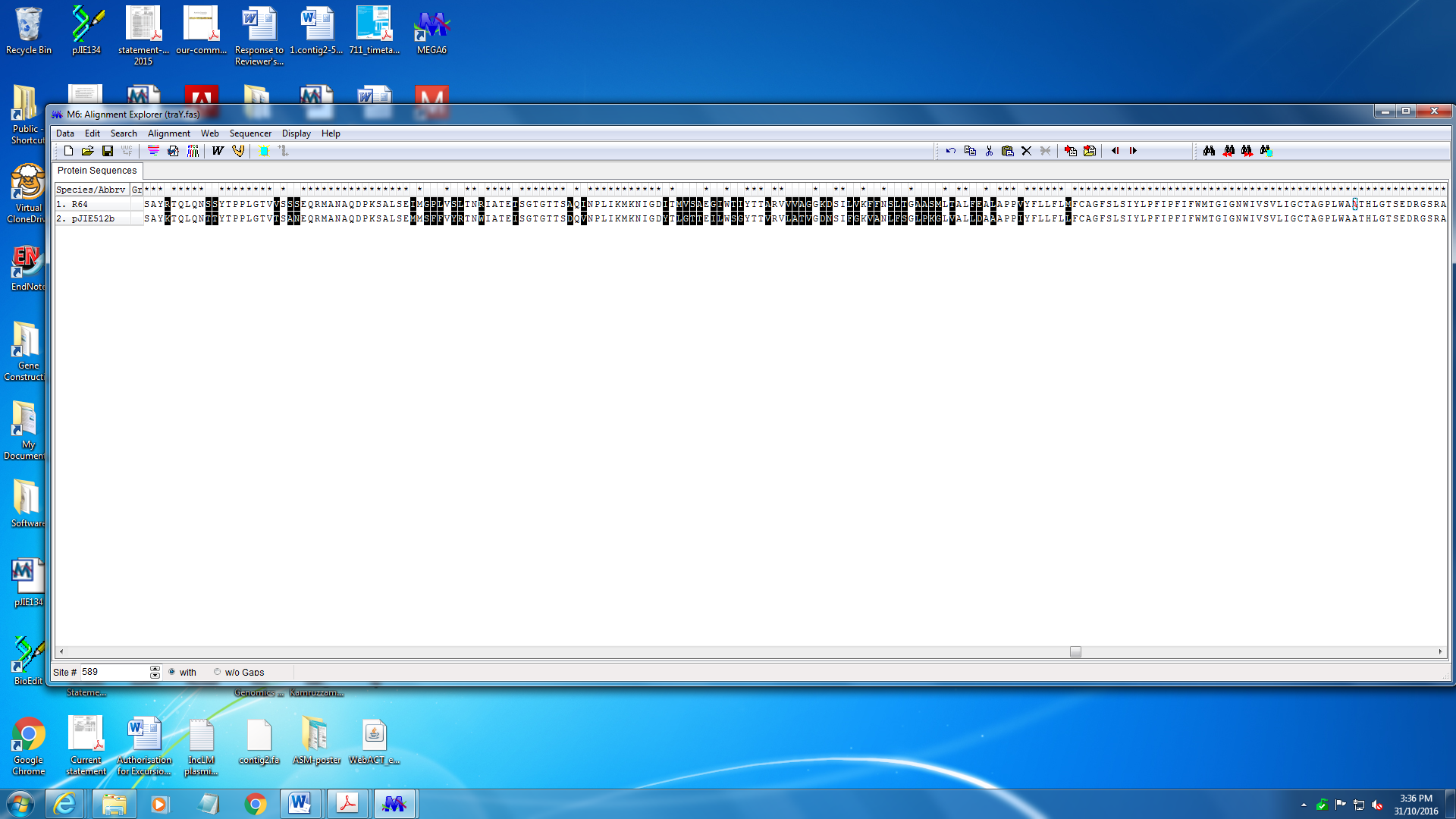


**A**


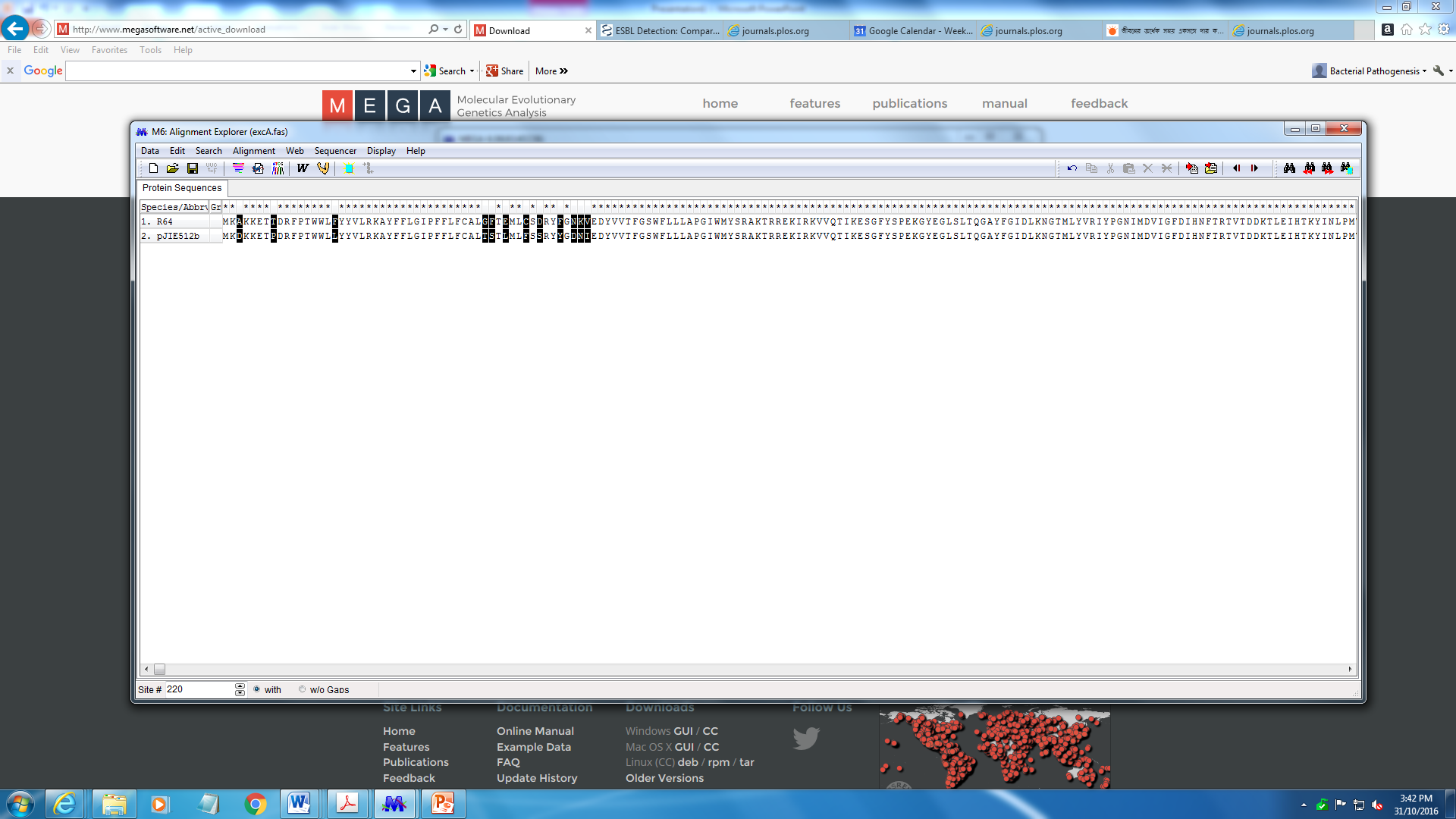

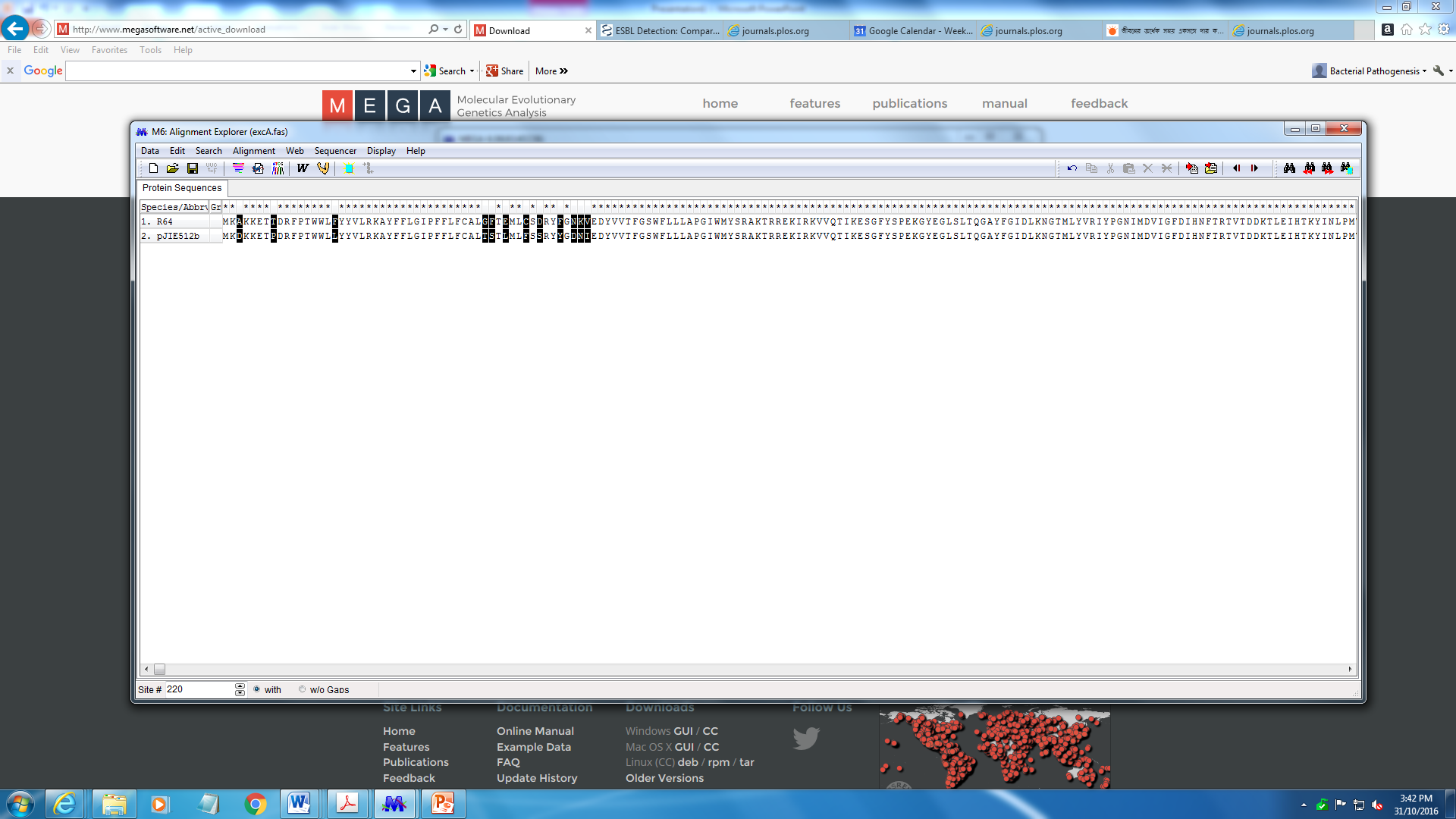


**B**

412

549

430

522

1

61
